# Application of the P300 Event-Related Potential in the Diagnosis of Epilepsy Disorder: A Review

**DOI:** 10.3390/scipharm86020010

**Published:** 2018-03-26

**Authors:** Kandhasamy Sowndhararajan, Minju Kim, Ponnuvel Deepa, Se Jin Park, Songmun Kim

**Affiliations:** 1 School of Natural Resources and Environmental Sciences, Kangwon National University, Chuncheon 24341, Korea; sowndhar1982@gmail.com (K.S.); camin1121@gmail.com (M.K.); taanishadeepa@gmail.com (P.D.); sejinpark@kangwon.ac.kr (S.J.P.); 2 Gangwon Perfume Alchemy Ltd., Co., Chuncheon 24341, Gangwon-do, Korea

**Keywords:** electroencephalography, epilepsy, event-related potential, oddball paradigm, P300

## Abstract

Epilepsy is one of the most serious chronical neurological disorders, affecting more than 50 million people worldwide. It can be defined as a spectrum disorder, and patients with epilepsy possess abnormalities in cognitive functions. A number of factors can cause cognitive dysfunctions in epileptic syndromes, including etiology, the age of onset, type of seizure and severity, duration, and antiepileptic drugs. Event-related potentials (ERPs) are very useful clinical and research instruments to evaluate cognitive function in patients with neuropsychiatry disorders. Event-related potentials directly reflect cortical neuronal activity and provide a particular level of temporal resolution. Among various ERP components, the P300 is the most important component for assessing cognitive processes such as attention, working memory, and concentration. Numerous studies have reported the abnormalities in amplitude or latency of P300 component of ERP in epileptic patients, and these abnormalities are indicative of cognitive dysfunction. Therefore, the purpose of this review is to consolidate the existing literature in connection with the use of P300 in epileptic patients.

## 1. Introduction

Electroencephalography (EEG) is one of the most effective electrophysiological examining techniques for understanding the neurobiological dysregulation. The EEG is a recording of fluctuating electrical waveforms attained from electrodes placed on the scalp of the human brain. The EEG signals arise from excitatory and inhibitory post-synaptic potentials in populations of pyramidal neurons of the cerebral cortex. The EEG is non-invasively used to diagnose epilepsy, cognitive disturbances, brain death, dementia, cerebrovascular brain disease, and other psychological diseases. The time-locked activity of EEG is known as event-related potential (ERP). Event-related potential is a non-invasive approach that represents any stereotyped electrophysiological response resulting from sensory, cognitive, or motor processes [1]. Event-related potentials are very small voltages generated in the brain structures in response to specific events or stimuli. Event-related potentials have been linked with different cognitive functions, such as attention, concentration, memory, and decision-making. Therefore, ERPs are considered as a clinically important tool for evaluating cognitive dysfunction in various neurological disorders, including Alzheimer’s, Parkinson’s, epilepsy, schizophrenia, and stroke [2,3,4].

Event-related potential components are characterized by their positive or negative polarity, latency, scalp distribution, and relation to experimental variables. The important ERP components are P50, N100, P200, N200, P300, N300, P400, N400, P600, mismatch negativity, contingent negative variation, and movement-related cortical potentials [5]. The sequence and latencies of ERP components track the time course of processing activity in milliseconds, while amplitudes of ERP exhibit the extent of allocation of neural resources to particular cognitive processes [2]. Among them, P300 has been widely used, with endogenous potential for evaluating cognitive functions in various neurophysiological disorders due to its very stable latency in normal controls [2,6]. A variety of paradigms have been used to elicit the P300, of which an oddball is the most commonly used paradigm. In the oddball paradigm, P300 is elicited when a subject detects an infrequent or target stimulus in a regular train of standard stimuli [7]. The P300 has a centro-parietal scalp distribution with its maximum over midline scalp sites. It is generally largest at parietal and central electrode sites with a peak time of about 250–500 ms after stimulus onset. The P300 has two important subcomponents, such as P3a, and the most commonly known P3b [8]. Variations in the latency, amplitude, and topography of the P300 highly associate with clinical findings in a broad range of disorders and brain damages. This P300 component has been mainly applied to study age-related cognitive dysfunctions, because it reflects attention and memory processes. In addition, this component has been used to study psychiatric diseases, including alcoholism, depression, and schizophrenia [7]. Among the various neurological disorders, epilepsy is one of the important causes of central auditory disorders, and the P300 component characterizes a neurophysiological index of attention-dependent auditory processing.

Epilepsy is a chronic neurological disorder, and it has a prevalence of 5 to 10 cases per 1000 people. The causes of epilepsy may be genetic or acquired, such as brain tumors or strokes [9]. Previous studies reported that abnormalities in cognition, behavior/psychiatric status, and psychosocial functioning in relation to patients with epilepsy show cognitive and behavioral disturbances, especially in terms of memory, attention, and mental processing speed. In patients with epilepsy, 44% had difficulties in learning and psychomotor retardation, and 59% had sleepiness or tiredness problems. In addition, 63% of patients are unable to achieve activities or goals due to antiepileptic drug (AED) therapies. Moreover, cognitive impairments are already present in more than 50% of newly diagnosed and untreated epileptic patients. A study indicated that 64.5% children with abnormal brain imaging had cognitive impairments (Intelligence Quotient, IQ < 80) [3,10,11]. Numerous electrophysiological alterations occur in epilepsy (temporal lobe epilepsy (TLE), idiopathic generalized epilepsy (IGE), benign childhood epilepsy with centrotemporal spikes (BCECT), etc.) that may have worth for its diagnosis, including changes in the P300 component of event-related potentials. The abnormalities in amplitude or latency of P300 are indicative of impairment in cognitive processing. In general, ERP abnormalities can be caused by various factors, including the epileptogenesis process itself, the frequency of seizures, lesions, and anti-epileptic treatments [12]. Furthermore, the P300 amplitude relates positively with memory ability in healthy individuals and decreases of P300 amplitude are linked with decreased brain activation and cognitive impairment [13]. A number of studies have reported the role of P300 component to diagnose epilepsy. Hence, the aim of this paper is to provide a review of available literature in relation to the use of P300 component in patients with epilepsy. The present review gives a summary of cognitive functions in epilepsy disorder using the P300 component.

## 2. The P300 Background

The P300 wave was first described by Sutton et al. in 1965. The P300 is a late component of ERP, and a positive deflection occurs at a latency of about 300 ms from after stimulus onset. It is an important ERP component for noninvasively evaluating cognitive functions in humans [14,15]. The oddball paradigm is the most common method to elicit P300 component. In this paradigm, the subject is instructed to detect target stimuli, and not to frequent stimuli. A main determinant of the P300 amplitude is the subjective probability of the rare stimuli. Objective probability and environmental rareness are other important factors [16,17]. The P300 waveform is a marker of cognitive activity that appears anywhere from 250 to 500 ms after the presentation of infrequent auditory, visual, or somatosensory stimulus [18]. It was reported that the P300 is commonly largest at parietal and central electrode locations [14,19]. The P300 consists of two functionally different components, such as P3a and P3b. The P3a has an anterior distribution and is typically elicited by repeated irregular non-target stimuli. The P3a is suggested to reflect stimulus-driven, or bottom-up, attentional orienting to a salient but irrelevant stimulus [8,20]. The P3b has a late latency with parietal scalp distribution and is elicited after the emergence of a rare target within a train of frequent irrelevant stimuli [8,21]. The P300 and its underlying subprocesses could reflect rapid neural inhibition of ongoing activity to enable transmission of stimulus/task information from frontal to temporal-parietal positions. P300 signals could arise from the initial need to increase focal attention during stimulus detection comparative to working memory contents [22,23]. The main generators of P300 component are the hippocampus, thalamus, and the mesencephalic reticular formation. In addition, some other reports suggest that the P300 may arise from multiple generators, such as cortical and subcortical structures [12,24]. The P300 component offers information about various cognitive processes, including memory, attention, auditory discrimination, processing of sequential information, and decision making [12]. This potential is associated with the time that a subject takes to process a given stimulus.

The P300 component is calculated by evaluating its amplitude and latency. The P300 amplitude (μV) can be expressed as the difference between the average pre-stimulus baseline voltage and the largest positive-going peak of the ERP waveform within a time window. However, the range can differ depending on stimulus modality, task conditions, the age of the subject, etc. The P300 latency (ms) can be expressed as the time from stimulus onset to the point of maximum positive amplitude within a time window [3,8,25]. The scalp distribution of P300 is defined as the amplitude change over the midline electrodes such as Fz, Cz, and Pz [26]. The P300 latency is believed to index classification speed, which is proportional to the time needed to detect and evaluate the target stimulus. The P300 waveform is highly influenced by the cognitive process, and it does not depend on the physical features of the sensory input [27]. The P300 is generally believed to reflect an update of activity in corticolimbic circuits during attention and working memory processes [28]. Therefore, variation in P300 amplitude is presumed to reflect the degree to which that information is processed. The P300 amplitude is related to the number of attentional resources dedicated to a given task and has been linked with superior cognitive performance [29]. In general, the P300 latency increases during the classification of stimulus becomes more difficult. Further, the P300 latency is negatively associated with cognitive functions in normal subjects, with shorter latencies linked with excellent cognitive performance [7,30].

## 3. P300 and Epilepsy

Table 1 shows the use of P300 component for the assessment of cognitive functions in patients with epilepsy. In the table, we presented information about the number of diseased and control subjects, location of electrodes, stimuli (Hz), and intensity (dB) in addition to the activity of P300 component.

### 3.1. In Adults

There are numerous types of epilepsy, each with different causes, symptoms, and therapies. The epilepsy disease can be classified as idiopathic, symptomatic, or cryptogenic. In these, TLE is the most common form of symptomatic epilepsy characterized by unprovoked focal seizures. The use of auditory P300 component was studied in patients with TLE and IGE. When compared with IGE patients and controls, significantly longer age-corrected P300 latencies were observed in patients with TLE. The epilepsy duration or clinical manifestation was not related to the P300 in the same epileptic disorder. Age-corrected P300 latencies were significantly prolonged in TLE patients with bilateral temporal EEG foci than unilateral focus at Cz region [31]. In addition, significantly prolonged P300 latencies were observed in patients with abnormal EEGs than in patients with normal EEGs [32]. Chen et al. [47] reported that the age-corrected P300 latencies were significantly prolonged in patients with TLE than in healthy controls. Rabinowicz et al. [34] found that the P300 amplitude was significantly reduced ipsilateral to the epileptic focus in patients with TLE and concluded that the scalp P300 exactly lateralizes the epileptogenic focus. Bocquillon et al. [56] hypothesized that temporal lobe dysfunction would change the P300 source locations in patients with TLE. In the long-term EEG monitoring, the ERP amplitude was decreased in postictal recordings (9 out of 10 patients with TLE) when compared with preictal recordings [49]. Rocha et al. [58] studied the repercussions of left TLE for patients with left mesial temporal sclerosis (LMTS) in connection with the P300. In LMTS subjects, a greater P300 latency and a lower P300 amplitude were observed when compared with controls with a significant difference at C3A1 and C4A2 sites. Further, the authors stated that it was difficult to determine laterality effect of P300 among affected and unaffected hemispheres.

The role of centrally recorded P300 in patients with mesial temporal sclerosis (MTS)-TLE was investigated by Artemiadis et al. [61]. When compared with the control group, a decrease of P300 amplitude and an increase of P300 latency were observed in patients with MTS. Watanabe et al. [60] found that P300 latencies were prolonged by the ylang-ylang fragrance in both patients with TLE and healthy subjects. The P300 amplitude was significantly reduced by the ylang-ylang fragrance in healthy subjects. The results revealed that the effect of ylang-ylang fragrance on the P300 may be inhibited by impaired higher-order olfactory processing in TLE patients. A significantly prolonged P300 latency was observed in BCECT when compared with normal controls [33]. Soysal et al. [44] investigated the P300 component in patients with cryptogenic partial epilepsy (CPE). The amplitudes of P300 were lower in CPE group when compared with control groups. There was no correlation between the prolongation of P300 latencies and the type, serum level of AED, and seizure control. In right-handed male patients with IGE, the latency of P300 was significantly correlated with reaction time duration than healthy controls [57]. In another study, the P300 was assessed in patients with idiopathic partial epilepsies (IPE), and symptomatic partial epilepsies (SPE) to understand the connection between the cognitive and EEG activities. The results revealed that the impact of EEG abnormalities (especially paroxysmal discharges) on the P300 latency is comparatively minor, and the cognitive impairment in IPE or SPE predominantly originates from the epileptogenic lesion [56]. The auditory P300 was also used to assess the connection between the cognitive function and clinical seizures in patients with partial epilepsies (IPE and SPE). Patients with SPE found to have significantly prolonged age-corrected P300 latency compared to patients with IPE. There was no relationship with the prolongation of the P300 latency and the frequency of seizure, seizure type, and seizure duration. The results suggested that the cognitive impairment in partial epilepsies may arise from epileptogenesis or other factors [39].

In epileptic groups, the degree of EEG slowing was associated with the prolongation of age-corrected P300 latency. Accordingly, the P300 latency was significantly prolonged in the patients with marked slowing than patients with no slowing [37]. Ford et al. [48] used visual and auditory oddball ERP paradigms to compare patients with schizophrenia and patients with epilepsy syndromes in addition to normal subjects. Auditory P300 amplitude was decreased in patients with schizophrenia, as well as patients with epilepsy interictal chronic schizophrenia-like features. The results suggested that the P300 amplitude appears to be more sensitive to schizophrenia-like characteristics. Another study indicated that the effect of P300 on fatigue was superior, but depression had no effect on P300 in patients with epilepsy. The results suggested that fatigue is strongly associated with cognitive functions and depression [52]. Duncan et al. [25] investigated the effect of P300 on information processing in patients with generalized epilepsy (absence type and complex partial seizures). A significant reduction of the P300 was observed in absence patient group when compared with healthy controls. Further, there was a significant reduction in the P300 amplitude on the visual continuous performance test in both groups of seizure patients than controls. However, P300 on the auditory continuous performance test was reduced only in patients with absence seizures. Ozmenek et al. [54] found that P300 latencies were longer in patients with epilepsy when compared with control subjects. Further, patients with idiopathic primary generalized epilepsy (IPGE) found to have longer P300 latencies than secondary generalized epilepsy (SGE). Soysal et al. [44] found that the latencies of P300 were longer in IGE patients, and the amplitudes of P300 were lower in IGE group when compared with control groups.

### 3.2. In Children

In children with epilepsy, the P300 and neuropsychological analyses were used to examine cognitive activities such as attention and immediate recall. Sunaga et al. [35] evaluated the cognitive function in children with IGE and TLE by using P300. In their study, P300 latencies were negatively correlated with age at Pz and Cz in control subjects. Significantly longer P300 latencies were observed in patients with IGE when compared with control subjects. The authors suggested that the mesencephalic reticular formation and thalamus may play important roles in the genesis of generalized epilepsy. In children with TLE or rolandic epilepsy, there was no significant difference in auditory P300 latency and amplitude [63]. Konishi et al. [36] examined the auditory P300 in patients with childhood epilepsies (SPE, IGE, and IPE). The latency of P300 was significantly longer in patients with epilepsies than in controls. In their study, the prolongation of P300 latency was greatest in patients with SPE, mild in those with IGE, and minimum in those with IPE. Abnormalities in the P300 latency were observed at all ages during childhood in patients with SPE. The shortening of P300 latency with age was comparatively minor in patients with epilepsies than in controls. These results suggested that the P300 latency prolongation exhibits characteristic alterations with age in each epileptic disorder. In another study, Turkdogan et al. [50] recorded visual and auditory ERPs in epileptic children with magnetic resonance imaging abnormalities. The authors suggested that epileptic activity, itself, leads to prolonged P300 components of auditory ERPs and visual ERPs. In addition, the presence of structural abnormality indicated by magnetic resonance imaging is not a predictor of ERPs abnormalities. Gokcay et al. [6] used the P300 and neuropsychological tests to examine cognitive functions in children with childhood epilepsy with occipital paroxysms (CEOP). The CEOP group was found to have significantly longer P300 latency when compared with the control group. In a recent study, the influence of auditory P300 in children with BCECT and TLE was studied by Casali et al. [12]. In their study, the P300 latency was significantly prolonged in children with TLE group when compared with control group. However, P300 latencies and amplitudes were not significantly different between BCECT and control groups.

### 3.3. Anti-Epileptic Drugs and Treatments

Some authors also investigated the role of P300 in the effect of treatments in epileptic patients. A study was carried out to investigate the relationship between cognitive function and anti-epileptic drug (CBZ) in patients with BCECT and healthy controls, using the auditory P300. A significantly prolonged P300 latency was observed in BCECT when compared with normal controls. During the course of therapy, the prolongation of P300 latency was greatest. Further, there was a significant positive correlation between the age-corrected latency of P300 and the serum concentration of CBZ [33]. Tumay et al. [59] investigated the effect of anti-epileptic drugs, levetiracetam (LEV) carbamazepine (CBZ), and sodium valproate (VPA) in patients with epilepsy. The results revealed that the P300 latency of LEV treatment was significantly shorter when compared with VPA and CBZ treatments. However, P300 latencies of these three groups were significantly longer than the control group. Another study indicated that the P300 was shorter in patients with epilepsy than in controls, but there was no change in the P300 component after the treatment of topiramate or VPA [55]. Kubota et al. [42] used the P300 component to examine cognitive functions in patients with epilepsy (medicated and unmedicated groups). The P300 latency and amplitude were not significantly varied in the unmedicated group as well as in the control group. However, P300 latencies were significantly prolonged in the medicated group when compared with the control group. Further, latency prolongation was positively correlated with the seizure frequency, the number of drugs and the blood concentrations of AEDs administrated. In another study, both latency and amplitude of P300 did not significantly differ in groups of seizures and chronic use of AEDs [27]. Ozmenek et al. [54] found that ERP parameters were not significantly different with monotherapy and polytherapy groups. In addition, variable effects on latencies of ERP were observed by AED subgroups. Therefore, latencies of P300 play a major role in the examination of subclinical cognitive impairment in patients receiving AED treatment. Triantafyllou et al. [32] also reported that shorter P300 latencies were noticed in patients receiving anticonvulsant monotherapy when compared with patients receiving a combination of anticonvulsant polytherapy. The P300 component was also used to investigate the effects of vagus nerve stimulation (VNS) on patients with epilepsy. De Taeye et al. [62] evaluated the effect of VNS on noradrenergic signaling in the brain via the P300 component. In VNS responders, the P300 amplitude was significantly increased at the parietal midline electrode. In another study, the P300 amplitude was significantly increased in responders and significantly decreased in non-responders. Furthermore, the authors suggested that non-midline electrodes play a major role in recording P300 features than midline electrodes [65].

## 4. Conclusions and Future Perspectives

Monitoring patients using electrophysiological and behavioral devices may be more effective in demonstrating cognitive abnormalities. ERPs are used to determine the cognitive performance, diagnosis, and effects of treatment in epileptic patients. In particular, a number of studies have reported that a positive correlation between the P300 component and epilepsy. Previous studies stated that cognitive impairments may arise from the epileptogenesis process, spreading from epileptic foci and the effects of AEDs. Based on the literature, the P300 component has been mainly used to assess the cognitive dysfunction in patients with TLE, followed by other symptomatic and idiopathic epilepsies. In addition, alterations in the P300 latency can vary according to the epileptic syndrome, time duration, and frequency of seizure. The results revealed that the P300 latency was significantly prolonged in patients with epilepsy. An important function of P300 is to give information useful for discriminating among subtypes of epileptic disorders. Further, the use of P300 component in epileptic patients could be beneficial in the early identification of affected patients and may help in the selection of suitable remedial measures by understanding the status of anti-epileptic drug treatments. Collectively, the published reports suggest that P300 may be clinically useful as an indicator of cognitive function, although its diagnostic efficacy is questionable. However, the stimulus intensity for the elicitation of P300 (40 dB to 100 dB) is highly varied according to the authors. Hence, it is necessary to standardize the intensity level in order to enhance the use of P300 in different types of epilepsy.

## Figures and Tables

**Table 1:** The use of P300 component for the assessment of cognitive functions in patients with epilepsy.

S. No.	No. of Diseased Subjects	No. of Control Subjects	Site	Reference Electrode	Frequent/Rare Stimuli (Hz)	Intensity (dB)	Activity	Reference
1	65 (39 TLE patients and 26 IGE patients)	28	Cz and Pz	A1 and A2	1000/2000	70	In TLE patients with bilateral temporal EEG foci, significantly prolonged latencies were observed at Cz.	[31]
2	68	30	F3, F4, F7, F8, T3, T4, T5, T6, C3, Cz, C4, P3, Pz, P4, O1 and O2	Cheeks	1000/2000	70	Patients with epilepsy had significantly prolonged latencies than controls. Latencies were significantly prolonged in patients with TLE compared to patients with IGE.	[32]
3	23	54	Pz	A2	1000/2000	65	The latency was significantly prolonged in BCECT than in healthy controls. The latency prolongation was greatest during the course of therapy. The latency was shorter with age despite of continuous CBZ therapy.	[33]
4	15	-	28 sites	A1 and A2	1000/2000	100	The amplitude was significantly attenuated ipsilateral to the epileptic focus.	[34]
5	50 (32 with IGE and 18 with TLE)	39	Cz and Pz	A1 and A2	1000/2000	70	The age-corrected latencies were significantly longer in patients with IGE than in controls. The age-corrected latencies were not significantly differed with TLE and controls, or IGE and TLE.	[35]
6	129	53	Pz	-	1000/2000	65	The latency was significantly longer in patients with epilepsies than in controls. The prolongation of latency was greatest in patients with SPE, mild in those with IGE, and minimum in those with IPE. Abnormalities of latency were observed at all ages during childhood in patients with SPE, and at older ages in patients with IGE.	[36]
7	90	63	Pz	-	1000/2000		The degree of EEG slowing was associated with the prolongation of age-corrected latency. The latency was significantly prolonged in the patients with marked slowing than patients with no slowing.	[37]
8	72 (26 with IPE, and 46 with SPE)	67	Pz	-	1000/2000	65	The impact of EEG abnormalities (especially paroxysmal discharges) on the latency was comparatively minor, and the cognitive impairment in IPE or SPE predominantly originates from the epileptogenic lesion.	[38]
9	72	67	Pz	-	1000/2000	65	Patients with SPE found to have significantly prolonged age-corrected latency than in patients with IPE. No relationship with the prolongation of the latency and the frequency of seizure, seizure type and seizure duration.	[39]
10	12 (6 epileptics whose mean IQ was 100 and 6 epileptics whose mean IQ was 52)	9	Fz, Cz and Pz	A1 and A2	1000/2000	50	Latencies and amplitudes were not significantly differed among the three groups.	[40]
11	50	38	Fz, Cz, C3 and Oz	A1 and A2	1000/2000		A positive correlation between prolongation of latencies and course of epilepsy. The latency was closely associated with arithmetic, digit symbol and picture arrangement.	[41]
12	46 unmedicated and 74 medicated patients	78	Fz, Cz, and Pz	A1 and A2	1000/2000	70	The latency and amplitude were not significantly varied in the unmedicated group as well as in the control group. The latencies were significantly prolonged in the medicated group than in the control group.	[42]
13	84 (55 TLE patients with and 29 without AHS)	-	-	A1 and A2	-	-	Limbic amplitudes were decreased on the side of the epileptogenic focus only in patients with AHS. Latencies were prolonged bilaterally in AHS patients. Amplitudes were reduced bilaterally in patients with left-sided AHS.	[43]
14	64 CPE patients and 52 IGE patients	-	Fz and Cz	A1 and A2	1000/8000	95	Latencies were longer in IGE patients, and amplitudes were lower in both CPE and IGE groups than in controls. No correlation between the prolongation of latencies and the type, AED serum level, and seizure control.	[44]
15	20	20	Cz and Pz	A1 and A2	1000/2000	70	Patients with epilepsy were found to possess significantly longer latencies and lower amplitudes.	[45]
16	108	32	Fz, Cz, Pz	A2	1000/2000	70	The latency was significantly prolonged in patients with symptomatic epilepsy than in patients without detectable brain lesions. The prolonged latency was significantly correlated with epilepsy duration, seizure frequency and antiepileptic treatment.	[46]
17	27 patients IGE and 13 patients with TLE	60	P3 and P4	A1 and A2	125/750	70	The age-corrected latencies were significantly prolonged in patients with TLE than in patients IGE and controls.	[47]
18	24	-	Fz and Pz	A1 and A2	500/1000	80	Auditory amplitude was decreased in patients with schizophrenia as well as patients with EPI-SZ. Delay in P300 was related to patients with epilepsy and EPI-SZ with the exception of patients with schizophrenia.	[48]
19	10	10	F3 F4 FC3 FC4 C3 C4 CP3 CP4 P3 P4	A1 and A2	1000	-	The ERP amplitude was decreased in postictal recordings (9 out of 10 patients with TLE) when compared with preictal recordings.	[49]
20	50	21	Fz, Cz, and Pz	A1 and A2	1000/8000	70	Epileptic activity, itself, leads to prolonged P300 components of auditory ERPs and visual ERPs.	[50]
21	120 (55 with partial seizures, 45 with generalized seizures and 20 with intractable seizures)	25	Cz	A1 and A2	1000/8000	95	Significantly longer latencies were observed in the intractable and partial groups. Delayed P300 latencies were not significantly correlated with epilepsy duration, frequency of seizure and cerebral imaging pathologies.	[51]
22	30	25	Fz and Cz	A1 and A2	1000/8000	95	P300 latency was significantly longer in the childhood epilepsy with occipital paroxysms group.	[6]
23	73	31	Cz and Fz	A1 and A2	1000 /2000	-	The effect of P300 on fatigue was superior, but depression had no effect on P300 in patients with epilepsy.	[52]
24	30	30	Fz, Czand Pz	A1 and A2	1000/2000	60	The latency and amplitude were not significantly differed in groups of seizures and chronic use of AEDs.	[27]
25	21	21	Cz and Fz	A1 and A2	1000/2000	90	The latency and amplitude were not significantly differed among the groups.	[53]
26	40 (9 with IPGE and 31 with SGE)	40	Fz, Czand Pz	A1 and A2	1000/2000	70	The latencies were longer in patients with epilepsy when compared to control subjects. Patients with IPGE had longer latencies than SGE.	[54]
27	30	-	C3, P3, C4 and P4	Nose tip	-	-	The P300 was shorter in patients with epilepsy than in controls, but there was no change after the treatment of topiramate or VPA.	[55]
28	10	-	Frontal, central, parietal and temporal	A1 and A2	1000/1500	90	Temporal lobe dysfunction would change the P300 source locations in patients with TLE.	[56]
29	9 with absence epilepsy and 13with complex partial epilepsy	10	Frontal (Fpz, F3, Fz, F4), central (C3, Cz, C4), parietal(P3, Pz, P4), and occipital (Oz)	A1 and A2	Low −600, medium −1050, or high −150	50	A significant reduction of the P300 was observed in absence patients group than in healthy controls. A significant reduction in the amplitude on the visual continuous performance test in both groups of seizure patients than in controls. P300 on the auditory continuous performance test was reduced only in patients with absence seizures.	[25]
30	35 (IGE)	20	Fz and Cz	-	1000/2000	90	The latency was significantly correlated with reaction time duration in patients with epilepsy.	[57]
31	12	12	C3A1, C3A2, C4A1 and C4A2	-	1000/1500	75	In LMTS subjects, a greater latency and a lower amplitude were observed at all sites than controls with a significant difference at C3A1 and C4A2 sites.	[58]
32	53	20	Cz and Pz	A1 and A2	1000/2000	50	The latency of patients receiving LEV was significantly shorter when compared with patients receiving VPA and CBZ. Latencies of these three groups were significantly longer than the control group.	[59]
33	14	14	Fz, Cz and Pz	Nose tip	1000/1050	-	Latencies were prolonged by the ylang-ylang aroma in both patients with TLE and controls. The amplitude was significantly reduced in controls.	[60]
34	16	43 (12 men)	Pz	A1 and A2	1000/2000	40	A decrease of amplitude and an increase of latency were observed in patients with MTS than in controls. There was no correlation between duration of disease and features.	[61]
35	20	-	CPz and AFz	CPz	1024	50	In VNS responders, the amplitude was significantly increased at the parietal midline electrode.	[62]
36	19	16	Fz	A1 and A2	1000/2000	75	No significant difference in the latency and amplitude between the groups.	[63]
37	20	16	Fz	A1 and A2	1000/2000	75	The latency was significantly prolonged in TLE patients than in control.	[12]
38	75	30	C3 and C4	A1 and A2	1000/2000	80	The prolongation of latency was declined in integral functions of the CNS and information processing mechanisms occurring in epilepsy.	[64]
39	18	-	Pz	CPz	-	50	The amplitude was significantly increased in responders and decreased in non-responders. Non-midline electrodes are better P300 biomarkers.	[65]

AEDs, antiepileptic drugs; AHS, Ammon’s horn sclerosis; BCECT, Benign childhood epilepsy with centrotemporal spike; CBZ, carbamazepine; CNS, central nervous system; CPE, cryptogenic partial epilepsy; EEG, electroencephalography; EPI-SZ, epilepsy interictal chronic schizophrenia-like features; ERP, event-related potential; IGE, idiopathic generalized epilepsy; IPE, idiopathic partial epilepsies; IPGE, idiopathic primary generalized epilepsy; LEV, levetiracetam; LMTS, left mesial temporal sclerosis; MTS, mesial temporal sclerosis; VPA, sodium valproate; SGE, secondary generalized epilepsy; SPE, symptomatic partial epilepsies; TLE, temporal lobe epilepsy; VNS, vagus nerve stimulation; IQ, intelligence quotient
